# Design and Processing Method for Doppler-Tolerant Stepped-Frequency Waveform Using Staggered PRF

**DOI:** 10.3390/s21196673

**Published:** 2021-10-08

**Authors:** Yan Zhang, Chunmao Yeh, Zhangfeng Li, Yaobing Lu, Xuebin Chen

**Affiliations:** Beijing Institute of Radio Measurement, Beijing 100854, China; zoo1881@163.com (Y.Z.); lzfcasic@163.com (Z.L.); luyaobing65@163.com (Y.L.); cxb1995xx@126.com (X.C.)

**Keywords:** Doppler tolerance, radar waveform design, staggered pulse repetition frequency (SPRF), sparse stepped-frequency waveform (SSFW), synthetic wideband signal

## Abstract

Stepped-frequency waveform may be used to synthesize a wideband signal with several narrow-band pulses and achieve a high-resolution range profile without increasing the instantaneous bandwidth. Nevertheless, the conventional stepped-frequency waveform is Doppler sensitive, which greatly limits its application to moving targets. For this reason, this paper proposes a waveform design method using a staggered pulse repetition frequency to improve the Doppler tolerance effectively. First, a generalized echo model of the stepped-frequency waveform is constructed in order to analyze the Doppler sensitivity. Then, waveform design is carried out in the stepped-frequency waveform by using a staggered pulse repetition frequency so as to eliminate the high-order phase component that is caused by the target’s velocity. Further, the waveform design method is extended to the sparse stepped-frequency waveform, and we also propose corresponding methods for high-resolution range profile synthesis and motion compensation. Finally, experiments with electromagnetic data verify the high Doppler tolerance of the proposed waveform.

## 1. Introduction

The wideband imaging ability is one of the most important functions of modern radar that may provide high-resolution information about targets. Wideband signals may be roughly divided into two categories, i.e., instantaneous wideband signals and the synthetic ones [1]. The instantaneous wideband signal achieves a wide bandwidth within a single pulse, which has the advantages of a short observation duration and a simple signal processing method. However, it has high requirements for hardware, which raises the cost of the radar system or even makes it unrealizable. The synthetic wideband signal, such as the stepped-frequency waveform (SFW), achieves a wide bandwidth by a sequence of pulses that is called a burst. Each burst has different carrier frequencies, and a wide bandwidth may be achieved by the synthetic processing method. In general, a synthetic wideband signal can be realized more easily for engineering practice due to its instantaneous narrow bandwidth.

The SFW has been used in radar systems [2,3], and different intra- and inter-sub-pulse modulation methods are applied. For instance, intra-sub-pulse modulation includes a single-frequency signal, a linear frequency modulation signal, a phase, or a frequency-coded signal [4,5], and inter sub-pulse modulation includes a linear frequency-stepping signal and a random or coded frequency-hopping signal [6,7]. In recent years, many studies on the SFW have been presented to improve radar imaging quality and anti-jamming ability, such as the compensation methods of system error [8,9], grating lobe suppression [10,11], and velocity compensation [12,13]. However, the duration of the SFW burst is too long, which leads to a reduction in detection efficiency and Doppler sensitivity, limiting its application to various scenes [14]. Therefore, to reduce the total duration, the sparse SFW (SSFW) has been designed for sparse target scenes [15]. Normally, the SSFW may reduce the number of sub-pulses without changing the synthesis bandwidth. Meanwhile, the sparse frequency band has a low probability of intercept (LPI), which improves the anti-jamming ability [16]. The research on the SSFW mainly includes waveform optimization, which may reduce the grating lobe caused by spectrum sparsity [17]. Further, methods of sparse basis functions’ adaptive optimization [18] as well as motion estimation and compensation [19] are proposed to attain better reconstructed performance.

Nevertheless, both the SFW and the SSFW are still Doppler sensitive. It means a small velocity compensation deviation will lead to a serious decrease in imaging quality. Thus, this characteristic greatly limits the application of the SFW. For this problem, the current research is carried out from two aspects. On the one hand, the velocity measurement compensation algorithm is studied. In [20], velocity measurement combines cross-correlation and range Doppler coupling. Meanwhile, a new algorithm was proposed based on the high-precision echo model in [21]. Based on the SSFW, the authors in [22] divide the translational motion into two parts, called the inter-pulse and the inter-burst translational motion, and they jointly design the cost function and estimate of motion parameters by using particle swarm optimization. In [23], two-dimensional motion compensation is achieved by the auto-focus algorithm in sparse reconstruction. On the other hand, the waveform design method is proposed. For instance, a new modulation format of chirp radars with stepped frequency is designed synthetically in [24] to remove range migration in detecting high-speed targets. In [25], it is shown that the complementary code is designed to eliminate the coupling relationship between range profile and velocity, thus improving the accuracy of velocity measurement. Then, the SFW with a different pulse repetition frequency (PRF) is designed for phase cancellation to eliminate the phase deviation caused by velocity [26,27]. However, the waveform design methods above focus on improving the accuracy of velocity or processing multiple bursts jointly but not on extending the Doppler tolerance directly in one burst of the SFW.

Doppler sensitivity is a common problem existing in the radar measurement for moving targets. Therefore, many researches are devoted to radar waveform design in order to extend the Doppler tolerance, such as the frequency and phase-coded waveform [28,29], piecewise modulation waveform [30] and hyperbolic frequency modulation waveform with Doppler invariance [31]. However, in practical applications, the frequency phase-coded waveform performs poorly due to the error in code switch, and the piecewise modulation waveform is actually an approximation of the ideal high-Doppler-tolerance waveform. Although the hyperbolic frequency modulation waveform has inherent Doppler invariance, the characteristic is only reflected in a single pulse. The proposed waveform is based on the SFW for high Doppler tolerance, which has fixed the frequency points and instead a staggered PRF (SPRF) [32,33,34,35,36]. Moreover, the modulation performance of the proposed waveform is similar to the hyperbolic frequency modulation waveform, and it is able to synthesize a high-resolution range profile (HRRP) with high quality.

The waveform design method is extended to the SSFW and the synthetic wideband signal by compressed sensing (CS). Nowadays, CS has developed a set of effective theories [37], which are widely used in the HRRP [38], SAR [39], and ISAR [40]. Especially in the air scene, the radar target is sparse relative to the observation window. Thus, we still get focusing results by CS when the spectrum of the waveform is incomplete [41]. In addition, electromagnetic data of an unmanned aerial vehicle (UAV) are used to verify the effectiveness of the proposed waveform design method [42]. Because the UAV has been widely used in life, disaster relief, and even war, and has become popular in the field of radar recognition, it is worth using these electromagnetic data to verify the proposed waveform [43,44].

## 2. Signal Model and Analysis

### 2.1. Generalized Echo Model of the SFW and SSFW

The CPRF-SFW transmits multiple sub-pulses with a stepped carrier frequency and constant PRF so as to improve the range resolution and synthesize wideband signals. However, the SPRF-SFW modulates the PRF to the staggered one. If the time component is expressed as the sum of fast time and slow time, $t=t_{s}+T_{i}$, then one burst of the transmitted SPRF-SFW signal is expressed as:

(1) $$s_{0}\left(i,t_{s}\right)=\sum_{i=0}^{M-1}u_{i}\left(t_{s}\right)\exp\left[j2\pi f_{i}\cdot \left(t_{s}+T_{i}\right)\right]$$

where $u_{i}\left(t\right)$ is the waveform structure of the sub-pulse and $f_{i}$ is the carrier frequency. $f_{i}=f_{0}+i\Delta f$ represents the linear frequency step mode. The initial carrier frequency is $f_{0}$, and the step frequency is Δf. One burst includes M sub-pulses, and the pulse repetition interval of each sub-pulse is $T_{r1}$, $T_{r2}$,…, $T_{rM}$. Thus, the transmitting time is presented as $T_{i}=\sum_{m=0}^{i}T_{rm}$. Specifically, when $T_{i}=iT_{r}$, it will change to the CPRF-SFW.

**Figure 1:**
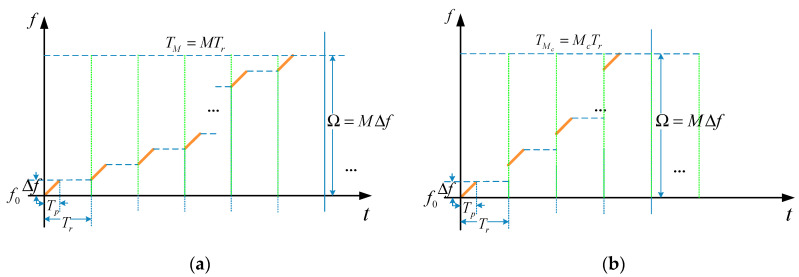

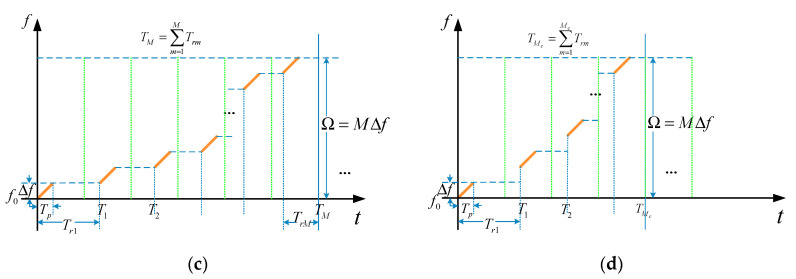
Time–frequency profile of (**a**) CPRF-SFW, (**b**) CPRF-SSFW, (**c**) SPRF-SFW, and (**d**) SPRF-SSFW.

Further, the SSFW only selects several frequencies for transmission compared with the SFW. $M_{c}$ is the number of selected sub-pulses. $f_{c(i)}$ is the selected carrier frequencies of the sub-pulse. Thus, the degree of spectrum integrity of the SSFW is defined as $\beta_{c}=M_{c}/M$. The higher the degree of spectrum integrity is, the more the sub-pulses are, and the SPRF-SSFW transmission signal is expressed as follows:

(2) $$s_{0}\left(i,t_{s}\right)=\sum_{i=0}^{M_{c}-1}u_{i}\left(t_{s}\right)\exp\left[j2\pi f_{c(i)}\cdot \left(t_{s}+T_{i}\right)\right]$$

where the selected carrier frequency is $f_{c(i)}=f_{0}+c(i)\Delta f$ and the selected mode is c(i)∈{0,1,2,…,M−1}, $i\in 0,1,2,\ldots ,M_{c}-1$. Specifically, when $T_{i}=iT_{r}$, it will change to the CPRF-SSFW [15]. The waveforms of the CPRF-SFW, CPRF-SSFW, SPRF-SFW, and SPRF-SSFW based on this model are presented in Figure 1, where $T_{p}$ is the sub-pulse width, $B_{p}$ is the sub-pulse bandwidth, and Ω is the synthetic bandwidth.

Moreover, the echo of the SPRF-SFW is analyzed. Suppose the initial distance between target and radar is $R_{0}$. The radial velocity of the target is defined as v, which is positive when the target is away from the radar. The velocity of the electromagnetic wave is c. Therefore, the two-way path delay under the initial distance is $\tau_{0}=2R_{0}/c$ and under the ith sub-pulse is $\tau_{i}=\tau_{0}+\left(2v/c\right)T_{i}$. Therefore, the base-band echo signal of the SPRF-SFW is expressed as:

(3) $$s_{rb}\left(i,t_{s}\right)=\sum_{i=0}^{M-1}u_{i}\left(t_{s}-\tau_{i}\right)\exp\left[-j2\pi f_{i}\left(2v/c\right)t_{s}\right]\exp\left(-j2\pi f_{i}\tau_{i}\right)$$

The model above is actually the generalized echo model of the SFW, which is composed of intra-pulse and inter-pulse terms. After constructing the model, the processing methods of the synthetic HRRP and the reasons of the Doppler sensitivity for CPRF waveforms are analyzed based on it.

### 2.2. Analysis of Doppler Sensitivity for CPRF Waveforms

The common signal processing method of the synthetic range profile is called twice pulse compression: one is intra-pulse compression, and the other is inter-pulse compression. The intra-pulse compression is to perform matched filtering on each sub-pulse, and the result of it is related to the waveform structure of a sub-pulse that can be a single-frequency, chirp and phase, or frequency-coded signal. Without loss of generality, we deduce the signal processing method with chirp, and the waveform structure of sub-pulse is expressed as:

(4) $$u_{i}\left(t\right)=\mathrm{rect}\left(\frac{t}{T_{p}}\right)\exp\left(j\pi \gamma t^{2}\right)$$

where $\mathrm{rect}\left(\frac{t}{T_{p}}\right)=\{\begin{array}{c} 1\\ 0 \end{array}\begin{array}{c} ,\\ , \end{array}\begin{array}{c} -T_{p}/2\leq t\leq T_{p}/2\\ \mathrm{others} \end{array}$ is the rectangular window function and $\gamma =B_{p}/T_{p}$ is the slope of sub-pulse chirp. Thus, according to Equation (3), the base-band echo is expressed as:

(5) $$s_{rb}\left(i,t_{s}\right)=\sum_{i=0}^{M-1}\mathrm{rect}\left(\frac{t_{s}-\tau_{i}}{T_{p}}\right)\exp\left[j\pi \gamma {\left(t_{s}-\tau_{i}\right)}^{2}\right]\exp\left[-j2\pi f_{i}\left(2v/c\right)t_{s}\right]\exp\left(-j2\pi f_{i}\tau_{i}\right)$$

Further, the obtained signal after intra-pulse compression is expressed as the structure of amplitude and phase, namely:

(6) $$y_{pi}\left(i,t_{s}\right)=\sum_{i=0}^{M-1}A\left(t_{s}\right)\cdot \exp\left(j\varphi \right)$$

(7) $$\begin{array}{c} A\left(t_{s}\right)=\sum_{i=0}^{M-1}\mathrm{rect}\left(\frac{t_{s}-\tau_{i}}{T_{p}}\right)\left|1-\left|\frac{t_{s}-\tau_{i}}{T_{p}}\right|\right|\\ \;\;\;\;\;\;\;\;\cdot \left|\mathrm{sinc}\left\{\pi \left[\left(-2v/c\right)f_{i}+\gamma \left(t_{s}-\tau_{i}\right)\right]\left(T_{p}-\left|t-\tau_{i}\right|\right)\right\}\right|\\ \cdot \exp\left[-j\pi \left(2v/c\right)f_{i}\left(t_{s}-\tau_{i}\right)\right] \end{array}$$

where sinc(x)=(sinx)/x and the inter-pulse phase term is $\varphi =-2\pi f_{i}\tau_{i}$. We find that the velocity of the target makes the peak of the intra-pulse compression shift from $\tau_{i}$ to $\tau_{i}+\left(2vf_{i}\right)/\left(c\gamma \right)$ and makes the peak value from 1 to $1-\left|\left(2vf_{i}\right)/\left(cB_{p}\right)\right|$. The results of intra-pulse compression are different in different waveform structures. Nevertheless, if the sub-pulse is a single-frequency signal, the bandwidth will be the reciprocal of the time width, which is not favorable for increasing the bandwidth and time width at the same time. Similarly, if the sub-pulse is a phase-coded signal, the result of pulse compression will be so sensitive to velocity that motion compensation would be difficult. Thus, the chirp signal is usually used in sub-pulse.

When the spectrum is complete, the phase term of the CPRF-SFW is:

(8) $$\varphi =-2\pi \left(f_{0}+i\Delta f\right)\left[\tau_{0}+\left(2v/c\right)iT_{r}\right]$$

The phase term, which includes the stationary phase caused by the radial distance of static target, is $\varphi_{r0}=-2\pi \left(f_{0}+i\Delta f\right)\tau_{0}$, including only the primary phase, but the motion phase caused by the velocity of the moving target is $\varphi_{v}=-2\pi \left(f_{0}+i\Delta f\right)\left(2v/c\right)iT_{r}$, including the primary phase and the quadratic phase. As a result, when inverse fast Fourier transform (IFFT) is used during inter-pulse compression, imaging defocusing occurs due to the existence of the quadratic phase term. In addition, the position of the target is $r_{p}=c\tau_{0}/2+vf_{0}T_{r}/\Delta f$ and the broadening is $r_{w}=2vMT_{r}$ which is obtained by the use of the stationary phase method.

Further, when the spectrum is incomplete, the phase term of the CPRF-SSFW is:

(9) $$\varphi =-2\pi \left(f_{0}+c(i)\Delta f\right)\left[\tau_{0}+\left(2v/c\right)iT_{r}\right]$$

The stationary phase is $\varphi_{r0}=-2\pi \left(f_{0}+c(i)\Delta f\right)\tau_{0}$, and the motion phase is $\varphi_{v}=-2\pi \left(f_{0}+c(i)\Delta f\right)\left(2v/c\right)iT_{r}$. Thus, when the CS algorithm is adopted during inter-pulse compression, the two phase terms above cannot be expressed by a unified basis, which leads to the defocusing of the synthetic HRRP.

In conclusion, the reason for Doppler sensitivity for the conventional CPRF waveform is the high-order phase component and filter mismatch caused by a moving target.

## 3. Design and Processing Method of SPRF Waveforms for High Doppler Tolerance

### 3.1. Waveform Design Method

After analyzing the Doppler sensitivity of CPRF waveforms, if the carrier frequencies of sub-pulses are determined, the Doppler tolerance may be improved by staggering the PRF of the sub-pulse.

By analyzing Equation (8), the design of the SPRF-SFW with high Doppler tolerance should meet the following two conditions.

Condition 1: The PRF of the sub-pulse is modulated so that the high-order phase is eliminated in $\varphi_{v}$. 

Condition 2: The pulse repetition interval (PRI), which is the reciprocal of the PRF, should have physical meaning that should be greater than zero. Thus, the condition 1 is expressed as:

(10) $$\varphi_{v}=-2\pi \left(f_{0}+i\Delta f\right)\left(2v/c\right)T_{i}=-2\pi \left(i\Delta f\right)\left(2v/c\right)\cdot k_{0}$$

where $k_{0}$ is a constant, which represents the weighting coefficient. Thus, Equation (10) is solved as:

(11) $$T_{i}=\frac{k_{0}\cdot i\Delta f}{f_{0}+i\Delta f}$$

At this time, the modulation mode of $T_{i}$ has met condition 1. Further, we give it physical meaning and connect it with the constant $T_{r}$ of the CPRF-SFW. Thus, we make $k_{0}=G_{0}T_{r}$, and the transmitting time of each sub-pulse $T_{i}$ is rewritten as:

(12) $$T_{i}=\frac{G_{0}\cdot i\Delta f}{f_{0}+i\Delta f}T_{r}$$

where $G_{0}$ is a waveform parameter, which determines the value range of the PRI.

As for condition 2, it is expressed as:

(13) $$T_{ri}=T_{i}-T_{i-1}>T_{r\min}>0$$

where $T_{r\min}$ is the minimum value of the PRI. We substitute the transmission time of each sub-pulse $T_{i}$ into Equation (13), and condition 2 is rewritten as:

(14) $$\frac{G_{0}f_{0}\Delta f}{\left(f_{0}+i\Delta f\right)\left[f_{0}+(i-1)\Delta f\right]}T_{r}>T_{r\min}>0$$

The SPRF-SFW under a negative frequency modulation slope is also designed by this method. Then, they are simplified to the following expression:

(15) $$\{\begin{array}{l} T_{i}=\frac{G_{0}\Delta f}{f_{0}+i\Delta f}iT_{r}\\ \begin{array}{cc} s.t. & G_{0}>0 \end{array} \end{array}\mathrm{or}\{\begin{array}{l} T_{i}=\frac{-G_{0}\Delta f}{f_{0}-i\Delta f}iT_{r}\\ \begin{array}{cc} s.t. & G_{0}<0 \end{array} \end{array}$$

**Figure 2:**
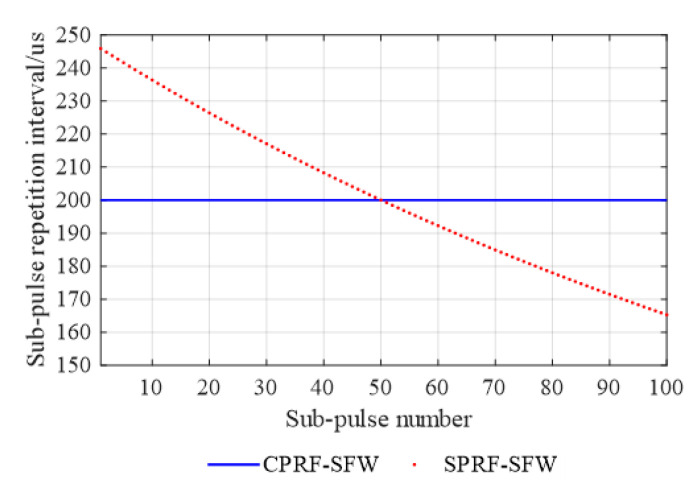
Pulse repetition interval of conventional CPRF-SFW and proposed SPRF-SFW with the waveform parameters shown in Table 1.

In addition, further analysis shows that when the waveform parameter $G_{0}<0$, the PRI of each sub-pulse is $T_{ri}=\frac{-G_{0}f_{0}\Delta f}{\left(f_{0}-i\Delta f\right)\left[f_{0}-(i-1)\Delta f\right]}T_{r}$, which is a monotonic increasing function. When the waveform parameter $G_{0}>0$, the PRI of each sub-pulse is $T_{ri}=\frac{G_{0}f_{0}\Delta f}{\left(f_{0}+i\Delta f\right)\left[f_{0}+(i-1)\Delta f\right]}T_{r}$, which is a monotonic decreasing function, as shown in Figure 2.

**Figure 3:**
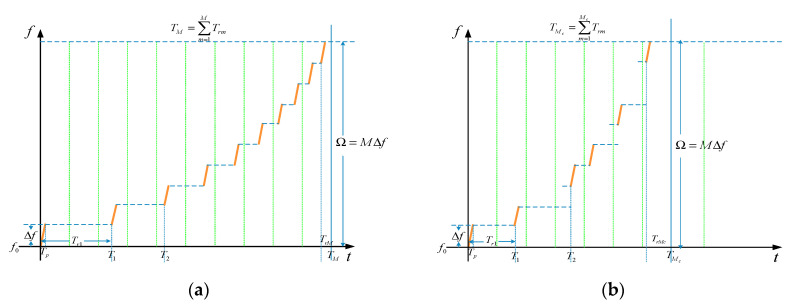
Time–frequency profile of (**a**) SPRF-SFW and (**b**) SPRF-SSFW designed by the proposed waveform method.

Further, when the spectrum is incomplete, the design of the SPRF-SSFW with high Doppler tolerance should also meet two conditions. Condition 2 remains unchanged, but Condition 1 changes to the following: the PRF of the sub-pulse is modulated so that the motion phase $\varphi_{v}$ and the stationary phase $\varphi_{r0}$ are expressed by a unified basis. Then, the transmitting time of each sub-pulse $T_{i}$ is expressed as:

(16) $$\{\begin{array}{l} T_{i}=\frac{G_{0}\Delta f\cdot c\left(i\right)T_{r}}{f_{0}+c\left(i\right)\Delta f}\\ \begin{array}{cc} s.t. & G_{0}\left[c(i)-c(i-1)\right]>0 \end{array} \end{array}$$

where c(i) is the coding law of the transmission frequency. When the waveform parameter $G_{0}>0$, c(i)>c(i−1), which is equal to the spectrum selection of positive frequency modulation coding. When the waveform parameter $G_{0}<0$, c(i)<c(i−1), which is equal to the spectrum selection of negative frequency modulation coding. The time–frequency profiles of waveforms designed by the proposed method using the SPRF are shown in Figure 3.

In conclusion, the waveform design method of SPRF waveforms with high Doppler tolerance is proposed. At the same time, as one of the staggered PRF waveforms, the SPRF-SSFW also has the characteristics of improving blind speed: the minimum blind velocity is increased from the constant PRF to the least-common-multiple PRF of each sub-pulse.

**Figure 4:**
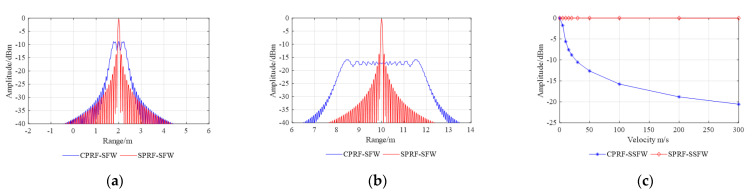
Comparison of imaging results under different waveforms at different velocities: (**a**) 20 m/s and (**b**) 100 m/s. (**c**) Curve of pulse compression gains versus velocities with the waveform parameters shown in Table 1.

In addition, Doppler tolerance is defined as the corresponding value when the pulse compression gain drops to the minimum allowable level, which is expressed as:

(17) $$\begin{array}{ccc} f_{DT} & s.t. & A_{peak}(f_{DT})/A_{peak}(0)=G_{DT} \end{array}$$

where $A_{peak}$ is the peak amplitude after pulse compression and $G_{DT}$ is the minimum allowable gain. Thus, the imaging results for different velocities and the curve of pulse compression gains versus velocities are shown in Figure 4.

Furthermore, by analyzing the echo model of the CPRF-SFW and SPRF-SFW, we get the ambiguity function of the synthetic wideband signal by twice pulse compression as:

(18) $$\chi \left(\tau ,v\right)=\int_{-\infty }^{+\infty }s\left(t\right)s^{*}\left[\left(1-2v/c\right)\left(t-\tau \right)\right]dt$$

**Figure 5:**
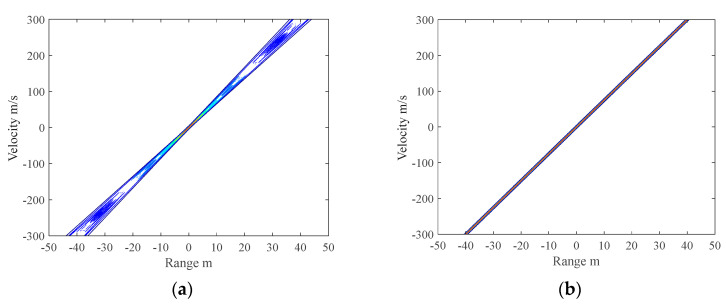
Ambiguity functions of (**a**) conventional CPRF-SFW and (**b**) proposed SPRF-SFW.

The contour maps of the ambiguity function for the two kinds of SFW, which have the same waveform parameters, after wideband synthesis are shown in Figure 5. Figure 5a is the ambiguity function of the CPRF-SFW, and Figure 5b is the ambiguity function of the SPRF-SFW. Both of them have range velocity coupling. However, when the velocity deviates, the broadening of the ambiguity function of the CPRF-SFW is obvious, which is the reason for the defocusing synthetic HRRP. In contrast, the ambiguity function of the proposed SPRF-SFW has no broadening, which means high Doppler tolerance.

### 3.2. Synthetic HRRP and Motion Compensation Process

**Figure 6:**
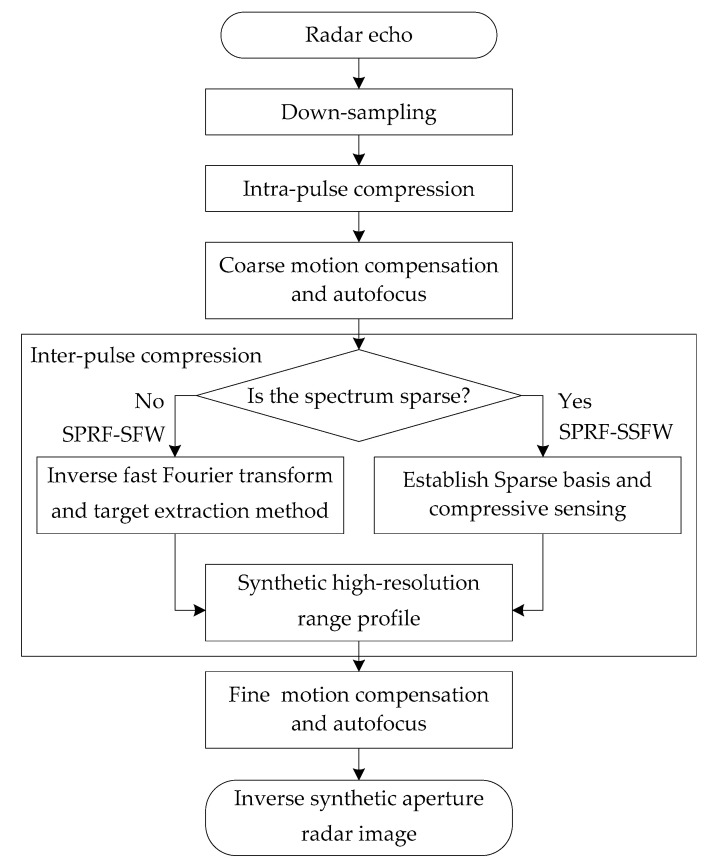
The flowchart of processing the proposed waveform.

The synthetic HRRP for the proposed SPRF-SSFW with high Doppler tolerance is also processed by twice pulse compression, as shown in Figure 6. Because the SPRF is only one inter-pulse modulation way, the intra-pulse compression is still the same for CPRF waveforms.

First, when the spectrum is complete, the phase term of the proposed SPRF-SFW is expressed as:

(19) $$\varphi =-2\pi \left(f_{0}+i\Delta f\right)\left[\tau_{0}+\left(2v/c\right)\frac{G_{0}\Delta f}{f_{0}+i\Delta f}iT_{r}\right]$$

The phase term includes the stationary phase $\varphi_{r0}=-2\pi \left(f_{0}+i\Delta f\right)\tau_{0}$ and the motion phase $\varphi_{v}=-2\pi \left(i\Delta f\right)\left(2v/c\right)G_{0}T_{r}$. Thus, none of them contains a quadratic or high-order phase term. The inter-pulse compression by IFFT is expressed as:

(20) $$\begin{array}{cc} y_{2}(\tau ) & =A\left(t_{s}\right)\cdot \int_{-\infty }^{\infty }\exp\left\{-j2\pi \left(i\Delta f\right)\left[\tau_{0}+\left(2v/c\right)G_{0}T_{r}\right]\right\}\\ & \cdot \exp\left(-j2\pi f_{0}\tau_{0}\right)\exp\left(j\cdot i\cdot \tau \right)di \end{array}$$

In this case, the range profiles without defocusing are obtained, and the target position is $r_{p}=c\tau_{0}/2+vG_{0}T_{r}$.

Then, when the spectrum is incomplete, the phase term of the proposed SPRF-SSFW is expressed as:

(21) $$\varphi =-2\pi \left(f_{0}+c(i)\Delta f\right)\left[\tau_{0}+\left(2v/c\right)\frac{G_{0}\Delta f}{f_{0}+c\left(i\right)\Delta f}c\left(i\right)T_{r}\right]$$

The phase term includes the stationary phase $\varphi_{r0}=-2\pi \left(f_{0}+c(i)\Delta f\right)\tau_{0}$ and the motion phase $\varphi_{v}=-2\pi \left[c\left(i\right)\Delta f\right]\left(2v/c\right)G_{0}T_{r}$. Thus, the two phase-terms above are expressed by a unified basis.

The sparse characteristics of the target are used in processing of the SPRF-SSFW, and the CS algorithm is proposed to transform the target imaging into a parameter estimation optimization problem. The measurement process is expressed as:

(22) Y=AX=ΦΨ⋅X

where the matrix is expressed in bold, $Y={\left[y_{1},y_{2},\cdots ,y_{M_{c}}\right]}^{T}$ is the measurement value vector, and $y_{i}$ is the echo of the i-th sub-pulse. $X={\left[x_{1},x_{2},\cdots ,x_{M_{d}}\right]}^{T}$ represents the target to be reconstructed, and $x_{j}$ is the amplitude of the i-th resolution unit. A is the sensing matrix with $M_{c}\times M_{d}$, Φ is the measurement matrix with $M_{c}\times M_{c}$, and Ψ denotes the basis matrix with $M_{c}\times M_{d}$. The target is sparse, which means that most of the elements in matrix X are zero or minimum. Thus, the solution of the measurement process is transformed into the $l_{0}$ norm optimization problem as follows:

(23) $$\begin{array}{ccc} \begin{array}{cc} \min & {\left\|X\right\|}_{0} \end{array} & s.t. & Y=AX \end{array}$$

The $l_{0}$ norm optimization problem is a nondeterministic polynomial problem. Thus, in the process of optimization, it is usually relaxed to an $l_{1}$ norm optimization problem. At the same time, considering the influence of weak scattering points and noise, the final optimization problem is expressed as follows:

(24) $$\hat{X}={\begin{array}{ccc} \min{\left\|X\right\|}_{1} & s.t. & \left\|Y-AX\right\| \end{array}}_{2}\leq \varepsilon$$

where ε represents one minimum value, the sensing matrix A=ΦΨ, and the measurement matrix Φ is usually set as a Gaussian random matrix, which expresses the influence of thermal noise in a single channel of the radar receiver. According to Equation (21), the elements of the basis matrix designed for the SPRF-SSFW, Ψ, are as follows:

(25) $$\Psi_{i,k}=\exp{\left[-j2\pi c(i)\Delta f\cdot kr_{x}\right]}^{T}$$

where $r_{x}=c/(2\Omega )$ is the synthetic range resolution and Ω is the synthetic bandwidth.

**Figure 7:**
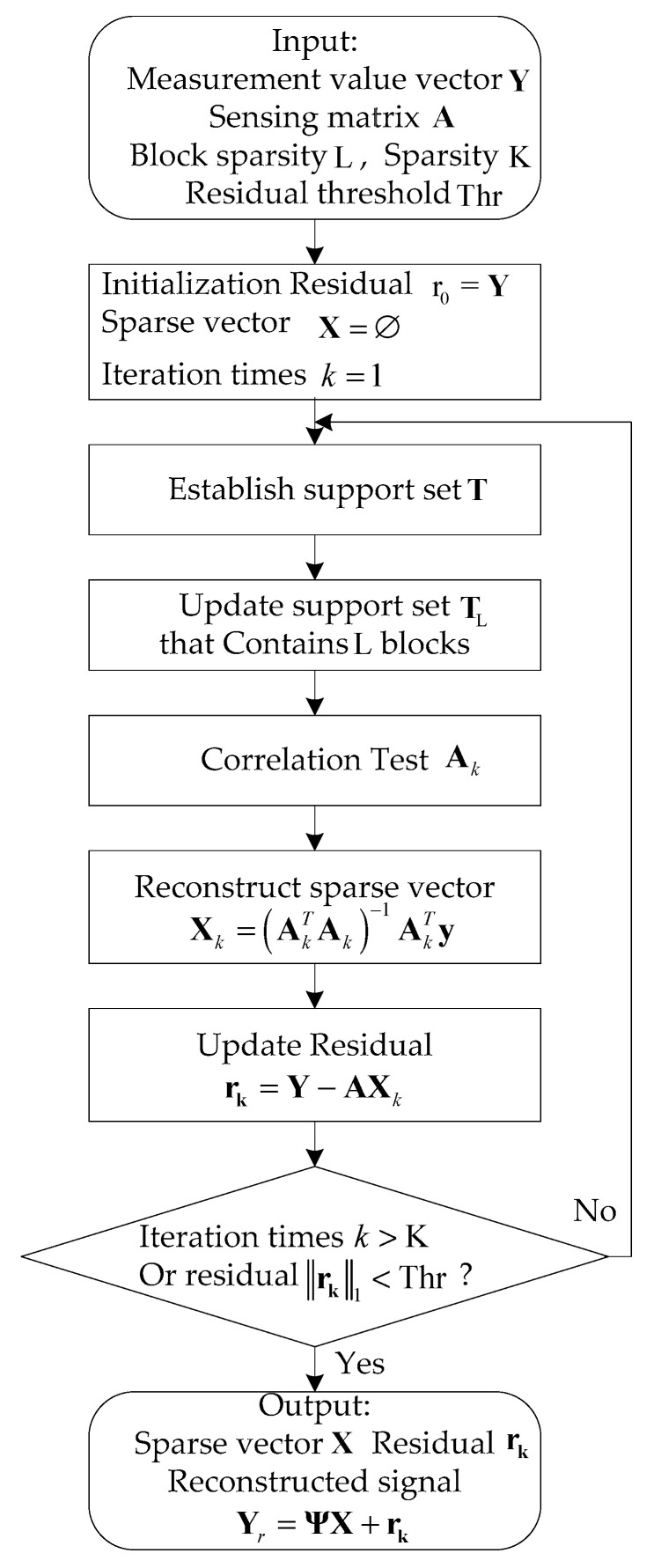
The flowchart of the joint-block sparse CS algorithm.

As for the optimization method, greedy algorithms, such as orthogonal matching pursuit, are used to solve this problem. CS methods have made great progress, and to improve the reconstruction efficiency, we use the joint-block sparse method for reconstruction [37,38]. The flowchart of the joint-block sparse CS algorithm is shown in Figure 7.

According to the analysis, the proposed SPRF waveform has high Doppler tolerance and has a focusing range profile of the moving target. However, the migration of the range profile still appears, which is not conducive to the accumulation of multi-frame imaging. Therefore, it is necessary to carry on the correlation velocity measurement and motion compensation.

The echo motion compensation of the SPRF waveform is similar to that of the CPRF [21,22], which usually consists of two steps: intra-pulse range alignment and inter-pulse phase compensation, as shown in Figure 6. It is necessary to go on the intra-pulse range alignment when the echo envelope movement cannot be ignored in a single frame. It effectively accumulates the intra-pulse compression results in the burst, reduces the loss of amplitude, and obtains the synthetic HRRP effectively. The envelope alignment step should ensure that the envelope movement caused by the residual velocity error is less than half of the sub-pulse resolution, which is expressed as $\left|v_{e}T_{M}\right|<c/4B_{p}$, namely:

(26) $$\left|v_{e}\right|<\frac{c}{4B_{p}T_{M}}$$

The requirement of intra-pulse range alignment is not high, which is called coarse motion compensation. Generally, intra-pulse range alignment is attained by system measurement or intra-pulse correlation velocity measurement [21]. After intra-pulse envelope alignment, the target is located in the same range resolution cell, and inter-pulse compression is performed.

In inter-pulse phase compensation, we obtain the motion parameters by the difference in the range profile. In the radar system, the difference in the synthetic range profile is obtained by the adjacent correlation method, and the velocity of the target is further obtained as $v=\Delta R_{p}/T_{M}$. The velocity measurement error should meet the requirement that the range difference is not greater than the resolution of the synthetic range profile c/2Ω, and then the velocity measurement error is expressed as:

(27) $$\left|v_{e}\right|<\frac{c}{2\Omega T_{M}}$$

The requirement of inter-pulse phase compensation is high, which is called fine-motion compensation [22]. Furthermore, the difference in the proposed SPRF waveform is more accurate than the CPRF waveform due to the synthetic range profile without defocusing. After processing, the estimated motion parameters of the target are obtained.

Furthermore, it is necessary to consider the influence of the target’s acceleration on motion compensation [23]. If the acceleration of the target is defined as a and the two-way path delay is $\tau_{i}=\tau_{0}+\left(2v/c\right)T_{i}+\left(a/c\right)T_{i}{}^{2}$, the base-band signal with uniform acceleration is presented as:

(28) $$\begin{array}{ll} s_{rb}\left(i,t_{s}\right) & =\sum_{i=0}^{M-1}u_{i}\left(t_{s}-\left[\tau_{0}+\left(2v/c\right)T_{i}+\left(a/c\right)T_{i}{}^{2}\right]\right)\\ & \cdot \exp\left\{-j2\pi f_{i}\left[\tau_{0}+\left(2v/c\right)T_{i}+\left(a/c\right)T_{i}{}^{2}\right]\right\} \end{array}$$

Further, the motion compensation term is expressed as:

(29) $$\begin{array}{cc} s_{p}\left(i,t_{s}\right) & =\sum_{i=0}^{M-1}u_{i}\left(t_{s}-\tau_{p}\right)\cdot u_{i}{}^{*}\left(t_{s}-\left[\tau_{p}+\left(2v_{p}/c\right)T_{i}+\left(a_{p}/c\right)T_{i}{}^{2}\right]\right)\\ & \cdot \exp\left\{j2\pi f_{i}\left[\left(2v_{p}/c\right)T_{i}+\left(a_{p}/c\right)T_{i}{}^{2}\right]\right\} \end{array}$$

where $v_{p}$ represents the estimated velocity, $a_{p}$ represents the estimated acceleration, and $\tau_{p}$ denotes the peak position after intra-pulse compression, used as the reference time delay. Thus, the SPRF-SSFW base-band echo after accurate motion compensation is expressed as:

(30) $$\begin{array}{ll} s_{pc}\left(i,t_{s}\right) & =s_{rb}\left(i,t_{s}\right)\cdot s_{p}\left(i,t_{s}\right)\\ & =\sum_{i=0}^{M-1}u_{i}\left(t_{s}-\tau_{p}\right)\cdot u_{i}{}^{*}\left(t_{s}-\left[\tau_{e}+\left(2v_{e}/c\right)T_{i}+\left(a_{e}/c\right)T_{i}{}^{2}\right]\right)\\ & \cdot \exp\left[-j2\pi f_{i}\tau_{0}\right]\cdot \exp\left[-j2\pi f_{i}\left(2v_{e}/c\right)T_{i}\right]\cdot \exp\left[-j2\pi f_{i}\left(a_{e}/c\right)T_{i}{}^{2}\right] \end{array}$$

where $v_{e}=v_{p}-v_{0}$ represents the residual velocity, $a_{e}=a_{p}-a_{0}$ represents the residual acceleration after motion compensation, and $\tau_{e}=\tau_{p}-\tau_{i}$ is the two-way delay of the difference between the reference point and each scattering point. The result shows that to ensure that the defocusing caused by acceleration is ignored, it is necessary to meet the following requirements:

(31) $$\left|a_{e}\right|\leq \frac{c}{2T_{M_{c}}{}^{2}f_{0}}$$

In addition, $\tau_{e}$ is small and the phase term related to $\tau_{e}$ is approximately constant, so the results of the SPRF-SSFW base-band echo after motion compensation are consistent with the results for a stationary target.

## 4. Experiment and Discussion

In this section, three simulation experiments combined with the electromagnetic data of the UAV are presented to verify the effectiveness of the proposed waveform design method. First, the proposed SPRF-SFW is compared with the conventional CPRF-SFW in order to verify the high-Doppler tolerance of the staggered PRF waveform. Second, the proposed SPRF-SSFW is designed and processed. On the one hand, it is compared with the CPRF-SSFW to verify the high Doppler tolerance. On the other hand, it verifies the effectiveness of the processing algorithm based on CS. Third, the velocities of target and waveform parameters are changed to verify the effectiveness of the waveform design method for high Doppler tolerance under different conditions.

**Figure 8:**
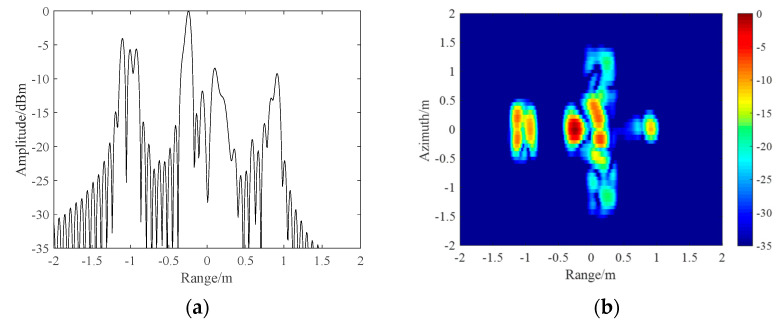
Ideal radar image using UAV electromagnetic data: (**a**) HRRP and (**b**) ISAR.

In addition, the ideal HRRP and ISAR images obtained from the electromagnetic data of the UAV at a certain angle are shown in Figure 8 and are used as benchmarks to compare with the imaging results of the designed waveform.

Further, the evaluation factors are set as the signal-to-noise ratio (SNR) and the main lobe width $W_{ML}$ for the HRRP image and the image entropy $H_{IE}$ and the image contrast $H_{IC}$ for the ISAR image. The SNR is defined as the ratio of the peak power of the pulse-compression-result-to-noise variance, which is expressed as:

(32) $$\mathrm{SNR}=\frac{{\left(A_{pot}\right)}^{2}}{\mathrm{var}\left(A_{st}:A_{pot-N_{p}},A_{pot+N_{p}}:A_{fh}\right)}$$

where $A_{pot}$ is the peak amplitude, $A_{st}$ is the amplitude of the starting point, $A_{fh}$ is the amplitude of the ending point, $N_{p}$ is the number of the protecting points around the target, and var represents the variance function. Thus, the higher the SNR is, the higher the imaging quality is.

The main lobe width was defined as the corresponding broadening at half of the peak, which is expressed as:

(33) $$\left\{ \begin{array}{l} W_{ML}=\left|x_{2}-x_{1}\right|\\ \begin{array}{cc} s.t. & A(x_{1})=A(x_{2})=A_{pot}/2 \end{array} \end{array}\right.$$

where $A(x_{i})$ is the amplitude of the range profile. Thus, the narrower the main lobe width is, the higher the imaging quality is.

The image entropy is expressed as:

(34) $$H_{IE}\left(x\right)=-\sum_{i=1}^{N}\left[P\left(x_{i}\right)\cdot \log_{2}P\left(x_{i}\right)\right]$$

where $P\left(x_{i}\right)$ is the amplitude of the imaging point. Thus, the lower the image entropy is, the higher the imaging quality is. In addition, according to [45], the image contrast is expressed as:

(35) $$H_{IC}\left(x\right)=\frac{\sqrt{\frac{1}{N}\sum_{i=1}^{N}{\left[P\left(x_{i}\right)-\frac{1}{N}\sum_{i=1}^{N}P\left(x_{i}\right)\right]}^{2}}}{\frac{1}{N}\sum_{i=1}^{N}P\left(x_{i}\right)}$$

Thus, the higher the image contrast is, the higher the imaging quality is.

### 4.1. Experiment I: SPRF-SFW

This subsection shows the HRRP and ISAR images to verify that the proposed SPRF-SFW has high Doppler tolerance. The design of the SPRF-SFW is carried out under the condition that the carrier frequencies obey the linear law with $f_{i}=f_{0}+i\Delta f$, and the same parameters of the SPRF-SFW and CPRF-SFW are shown in the Table 1. In addition, the target is the UAV electromagnetic data.

We further obtain the range resolution as 0.075 m, and the azimuth resolution is 0.2 m. To compare the SPRF-SFW with the CPRF-SFW, their burst durations are equal, that is, $T_{M}=MT_{r}$. Then, $\frac{G_{0}\Delta f}{f_{0}+M\Delta f}MT_{r}=MT_{r}$ is satisfied, and the waveform parameter $G_{0}$ is represented as:

(36) $$G_{0}=\left(f_{0}+M\Delta f\right)/\Delta f$$

**Figure 9:**
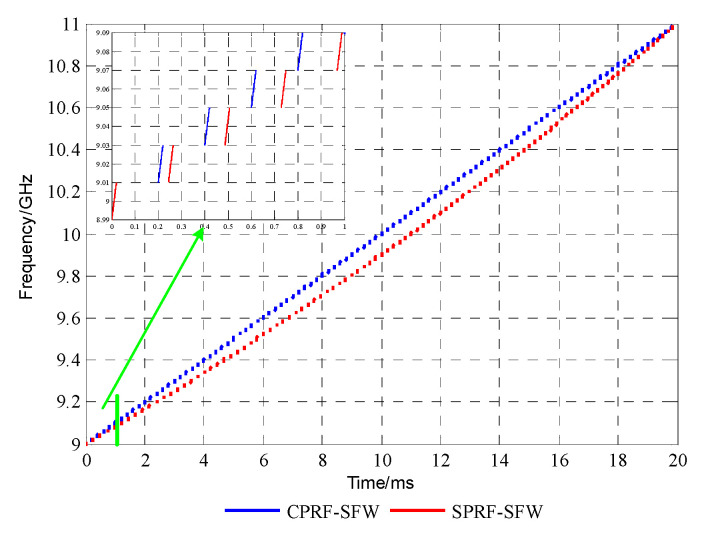
Time–frequency curves of conventional CPRF-SFW and proposed SPRF-SFW with simulation data.

Therefore, the PRI of the CPRF-SFW is 200 μs, and the PRI of the SPRF-SFW is obtained according to Equation (15), among which the maximum repetition interval is 245.92 μs, the minimum repetition interval is 165.29 μs, and other PRIs are shown in Figure 2. The time–frequency curves of the CPRF-SFW and the proposed SPRF-SFW under the designed parameters are presented in Figure 9.

**Figure 10:**
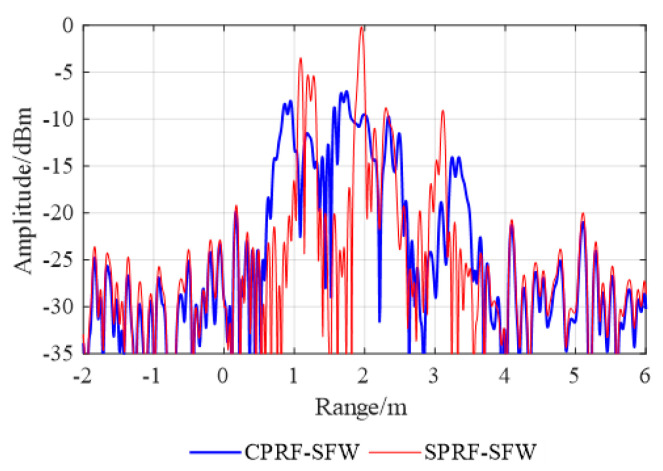
HRRP synthesis with conventional CPRF-SFW and proposed SPRF-SFW.

**Figure 11:**
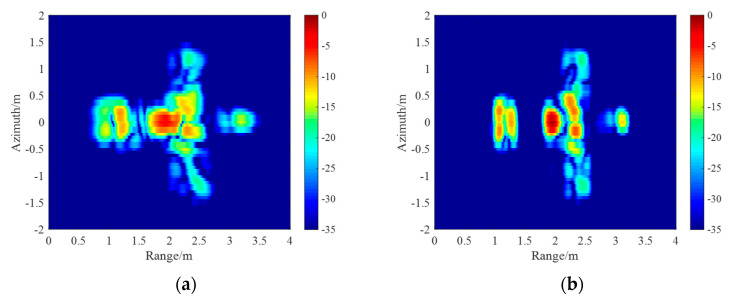
ISAR images with (**a**) conventional CPRF-SFW and (**b**) proposed SPRF-SFW.

The base-band signal of the SRPF-SFW with acceleration is discussed in the above waveform parameters. According to Equation (31), to ensure that the defocusing caused by acceleration is ignored, the residual acceleration after motion compensation should meet $\left|a_{e}\right|\leq 37.5\;{m/s}^{2}$. It is easy to be satisfied for aerodynamic targets. The residual velocity after coarse motion compensation is set as 20 m/s, which is far away from the radar direction, in order to better reflect the advantage of the proposed waveform. The received signal is contaminated by additive white complex Gaussian noise. Then, the SNR after intra-pulse compression is set as 16 dB, and the theoretical SNR is 26 dB after inter-pulse compression with 100 pulses. Therefore, the synthetic HRRPs of the conventional CPRF-SFW and the proposed SPRF-SFW are shown in Figure 10, and the ISAR images are shown in Figure 11.

As shown in Figure 10, the blue solid line is the synthesis HRRP of the CPRF-SFW and the red solid line is the synthesis HRRP of the SPRF-SFW. The imaging offset of the SPRF-SFW caused by the target motion is 2 m, which is consistent with the theoretical analysis. Additionally, compared with Figure 8a, it is observed that the CPRF-SFW imaging has both offset and defocusing caused by the target motion, with the SNR as 16.58 dB and the main lobe width as 0.71 m, while the SPRF-SFW imaging only has offset without broadening, with the SNR as 25.93 dB and the main lobe width as 0.08 m. For comparison, the SNR for the HRRP of the SPRF-SFW is higher and the main lobe width is narrower than that of the CPRF-SFW, which means that the imaging quality of the SPRF-SFW is higher than that of the CPRF-SFW for the same moving target.

The ISAR images of the CPRF-SFW and the SPRF-SFW are shown in Figure 11a,b, respectively. Compared with Figure 8b, the target motion causes defocusing of CPRF-SFW imaging, with the image entropy as 2484.08 bits and the image contrast as 2.32, while the proposed SPRF-SFW imaging is focused, with the image entropy as 1811.15 bits and the image contrast as 3.13. For comparison, the image entropy for ISAR images of the SPRF-SFW is lower and the image contrast is higher than that of the CPRF-SFW, which means that the imaging quality of the SPRF-SFW is higher than that of the CPRF-SFW for the same moving target.

Consequently, the above simulation results verify the high Doppler tolerance of the proposed SPRF-SFW, which makes up for the Doppler sensitivity of the CPRF-SFW, and has more practical value in complex scenes, especially in an imaging scene with multiple velocity targets such as target separation due to the difficulty in accurate motion compensation.

### 4.2. Experiment II: SPRF-SSFW

**Figure 12:**
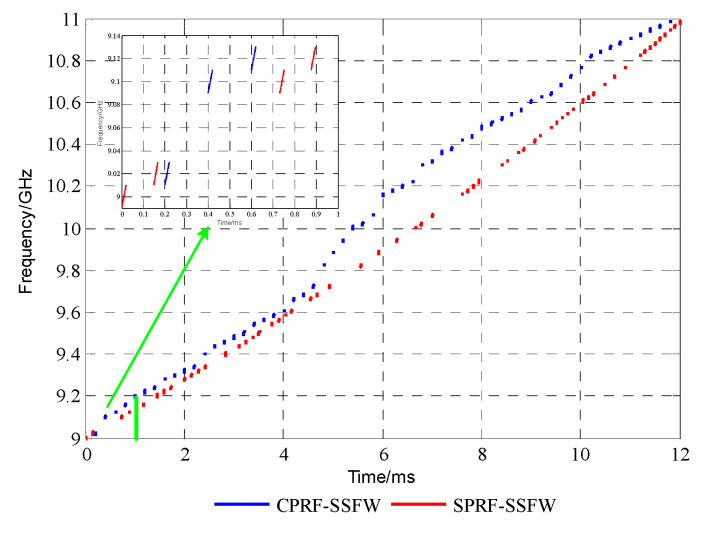
Time–frequency curves of conventional CPRF-SSFW and proposed SPRF-SSFW with simulation data.

In this subsection, the following simulation experiments are carried out when the spectrum is incomplete to verify that the SPRF-SSFW has high Doppler tolerance and obtains the wideband synthetic image by the CS algorithm. Therefore, the degree of the transmission spectrum integrity is set to 0.6. As a result, when compared with simulation experiment I, the number of sub-pulses is reduced from 100 to 60, the burst duration is reduced from 20 ms to 12 ms, and the carrier frequency of each sub-pulse is selected by random coding. Further, the PRI of the sub-pulse is calculated according to Equation (16), and other parameters are the same as those given in Table 1. The time–frequency curves of the CPRF-SSFW [15] and the proposed SPRF-SSFW under the designed parameters are presented in Figure 12.

**Figure 13:**
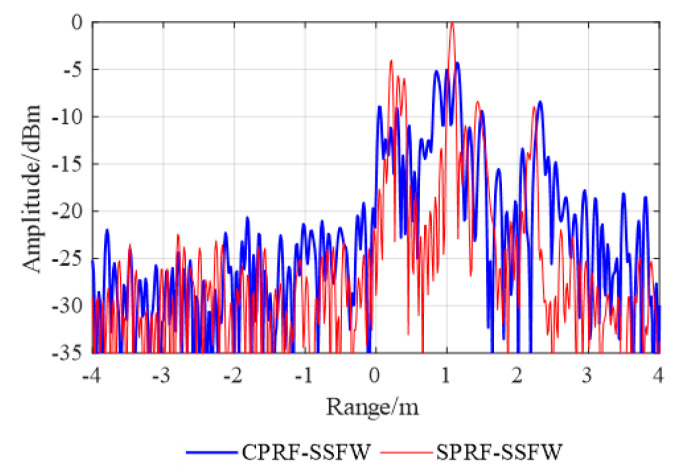
HRRP synthesis with conventional CPRF-SSFW and proposed SPRF-SSFW.

**Figure 14:**
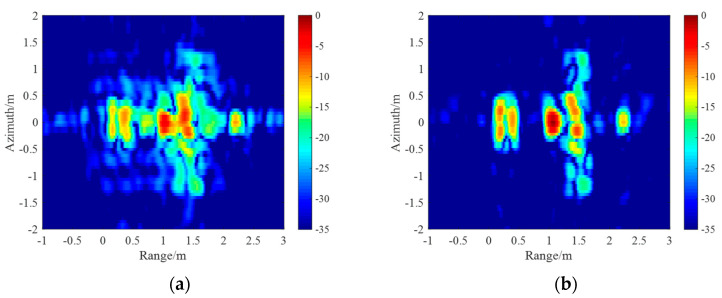
ISAR images with (**a**) conventional CPRF-SSFW and (**b**) proposed SPRF-SSFW.

Due to the incomplete spectrum, the burst duration of the SRPF-SSFW is lower than that of the SRPF-SFW in experiment I. Thus, the acceleration compensation tolerance of the SPRF-SSFW is higher. According to Equation (31), the residual acceleration after motion compensation should meet $\left|a_{e}\right|\leq 104.17\;{m/s}^{2}$. The residual velocity after coarse motion compensation is also set as 20 m/s. Meanwhile, the synthetic HRRP and ISAR images of the CPRF-SSFW and the SPRF-SSFW are shown in Figure 13 and Figure 14, respectively.

As shown in Figure 13, the blue solid line is the HRRP of the CPRF-SSFW and the red solid line is the HRRP of the SPRF-SSFW. The imaging offset caused by the target motion is 1.2 m, which is consistent with the theoretical analysis. Moreover, comparing these results with Figure 8a, we observe that the CPRF-SSFW imaging has both offset and defocusing caused by the target motion, with the SNR as 15.98 dB and the main lobe width as 0.39 m, while the SPRF-SSFW imaging only has offset without broadening, with the SNR as 23.95 dB and the main lobe width as 0.08 m. For comparison, the SNR for the HRRP of the SPRF-SSFW is higher and the main lobe width is narrower than that of the CPRF-SSFW, which means that the imaging quality of the SPRF-SSFW is higher than that of the CPRF-SSFW for the same moving target.

The ISAR images of the CPRF-SSFW and the SPRF-SSFW are shown in Figure 14a,b, respectively. Compared with Figure 8b, the target motion causes serious defocusing of CPRF-SSFW images, which is caused by the mismatch of the sensing basis, with the image entropy as 2712.49 bits and the image contrast as 2.05, while the SPRF-SSFW imaging has better focusing, and the sensing basis matches well, with the image entropy as 1947.65 bits and the image contrast as 2.96. For comparison, the image entropy for ISAR images of the SPRF-SSFW is lower and the image contrast is higher than that of the CPRF-SSFW, which also means that the imaging quality of the SPRF-SSFW is higher than that of the CPRF-SSFW for the same moving target.

Consequently, the above simulation results verify that the proposed SPRF-SSFW ha high Doppler tolerance and is processed by the CS algorithm well. Clearly, the SPRF-SSFW has a shorter burst duration and it is more suitable for a complex electromagnetic environment.

### 4.3. Experiment III: Different Conditions

In this subsection, the velocities of target and waveform parameters are changed, and the evaluation factors for ISAR images of conventional CPRF waveforms [15] (blue star line), optimized CPRF waveforms [11,17] (green cross line), and proposed SPRF waveforms (red diamond line) are further compared under different conditions in order to verify the effectiveness of the waveform design method for high Doppler tolerance.

**Figure 15:**
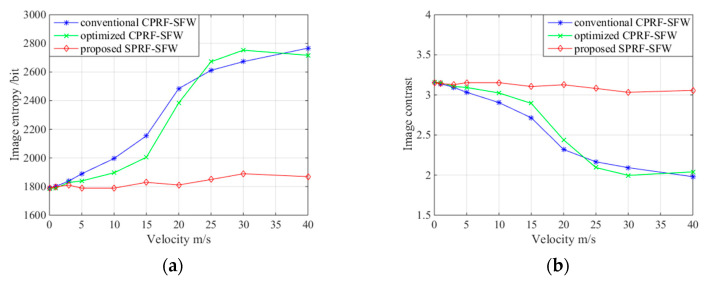
(**a**) Image entropy and (**b**) image contrast for ISAR images versus different velocities with conventional CPRF-SFW, optimized CPRF-SFW, and proposed SPRF-SFW.

First, when the spectrum is complete, the waveform parameters are fixed, as shown in experiment I, and the residual velocity of target changes. The imaging qualities of the conventional CPRF-SFW, optimized CPRF-SFW [11], and proposed SPRF-SFW are compared in Figure 15. The image entropy of the conventional CPRF-SFW increases and the image contrast decreases with velocity, which corroborates the decrease in imaging quality. The optimized CPRF-SFW increases the imaging quality in a certain velocity tolerance but decreases significantly when the velocity is too high. In addition, all evaluation factors of the proposed SPRF-SFW change little, which verifies the high Doppler tolerance for different velocities.

**Figure 16:**
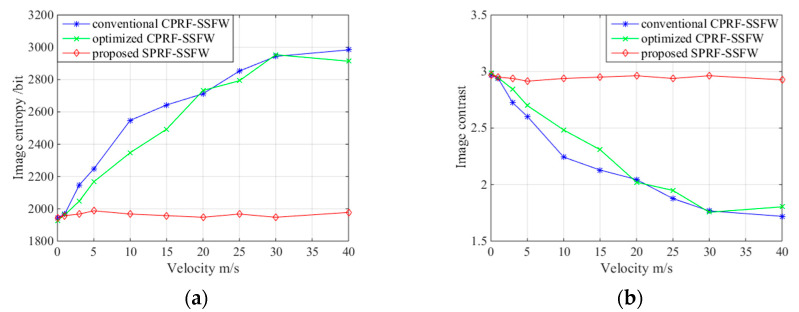
(**a**) Image entropy and (**b**) image contrast for ISAR images versus different velocities with conventional CPRF-SSFW, optimized CPRF-SSFW, and proposed SPRF-SSFW.

Then, when the degree of spectrum integrity is set to 0.6, the waveform parameters are fixed, as shown in experiment II, and the residual velocity of target also changes. The imaging qualities of the conventional CPRF-SSFW [15], optimized CPRF-SSFW [17], and proposed SPRF-SFW are compared in Figure 16. The image entropy of the conventional CPRF-SSFW increases and the image contrast decreases with velocity, which corroborates the decrease in imaging quality. The change is more rapid than that in Figure 15, because the imaging quality decreases more obviously when the echo of the moving target does not match the CS basis. The optimized CPRF-SSFW is similar to the conventional one, and the imaging focuses only when the velocity is low. In addition, all evaluation factors of the proposed SPRF-SSFW change little, and the imaging quality is stable, which also verifies the high Doppler tolerance for different velocities.

**Figure 17:**
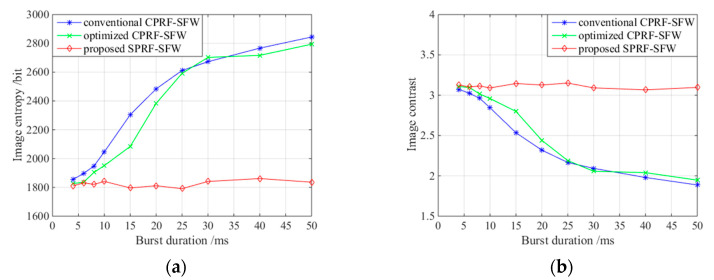
(**a**) Image entropy and (**b**) image contrast for ISAR images versus different burst duration with conventional CPRF-SFW, optimized CPRF-SFW, and proposed SPRF-SFW.

Next, when the spectrum is complete, the residual velocity of target is fixed as 20 m/s and the burst duration changes. The imaging qualities of the conventional CPRF-SFW, optimized CPRF-SFW [11], and proposed SPRF-SFW are compared in Figure 17. The image entropy of the conventional CPRF-SFW increases and the image contrast decreases with the burst duration, which verifies that the longer the burst duration, the lower the Doppler tolerance of the conventional CPRF-SFW. The optimized CPRF-SFW increases the imaging quality in a certain burst duration but decreases significantly when the burst duration is too long. In addition, all evaluation factors of the proposed SPRF-SFW change little, which verifies the high Doppler tolerance for different burst durations.

**Figure 18:**
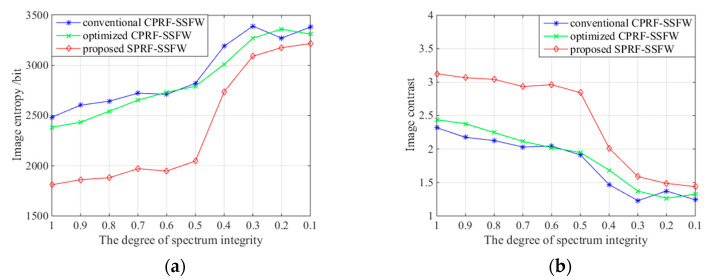
(**a**) Image entropy and (**b**) image contrast for ISAR images versus different degrees of spectrum integrity with conventional CPRF-SSFW, optimized CPRF-SSFW, and proposed SPRF-SSFW.

Finally, when the spectrum is incomplete, the residual velocity of the target is fixed as 20 m/s and the degree of spectrum integrity changes. The imaging qualities of the conventional CPRF-SSFW [15], optimized CPRF-SSFW [17], and proposed SPRF-SFW are compared in Figure 18. The image entropy of the conventional CPRF-SSFW is always higher and the image contrast is always lower, which indicates that the images of the moving targets defocus under various degrees of spectrum integrity. The optimized CPRF-SSFW has higher imaging quality than the conventional CPRF-SSFW but also has imaging defocusing. As a comparison, the image entropy of the proposed SPRF-SSFW increases slowly and the image contrast decreases slowly when the degree of spectrum integrity is bigger than 0.5. The main reason for the decrease in imaging quality is the decrease in the SNR, which is caused by the decrease in the accumulated pulse number. However, when the degree of spectrum integrity is smaller than 0.5, image entropy increases and image contrast decreases rapidly, which indicates that the degree of spectrum integrity should not be too low, otherwise the imaging quality will be affected. The experiment verifies the high Doppler tolerance of the proposed SPRF-SSFW for a high degree of spectrum integrity.

## 5. Conclusions

In this paper, we proposed a waveform design and a corresponding processing method using the SPRF to improve the Doppler tolerance of the SFW and the SSFW. Theoretical analysis and experiments verified that both the SPRF-SFW and the SPRF-SSFW designed by the proposed method have high Doppler tolerance and a focusing synthetic range profile. In addition, the SPRF-SSFW processed by CS has not only high Doppler tolerance but also a short burst duration and low probability of intercept, which improves the anti-jamming ability. Further work can expand the sub-pulse optimization design to improve the adaptability to maneuvering targets in a complex electromagnetic environment.

## Figures and Tables

**Table 1:** The same parameters of the SPRF-SFW and CPRF-SFW.

Parameters	Symbols	Value
Initial carrier frequency	$f_{0}$	9 GHz
Sub-pulse bandwidth	$B_{p}$	20 MHz
Sub-pulse width	$T_{p}$	20 µs
Synthesis bandwidth	Ω	2 GHz
Number of sub-pulses	M	100
Burst duration	$T_{M}$	20 ms
Sampling rate	$f_{s}$	40 MHz
Azimuth bandwidth	$B_{a}$	750 MHz

## Data Availability

The data presented in this study are available on request from the corresponding author.
